# 5G High Density Demand Dataset in Liverpool City Region, UK

**DOI:** 10.1038/s41597-025-06282-0

**Published:** 2025-12-10

**Authors:** Mukesh Kumar Maheshwari, Alessandro Raschellà, Michael Mackay, Max Hashem Eiza, Jon Wetherall, Jen Laing

**Affiliations:** 1 School of Computer Science & Mathematics, Liverpool John Moores University, Liverpool, L3 3AF UK; 2 Department of Electrical Engineering, Bahria University, Karachi Campus, Karachi, 75260 Pakistan; 3 CGA Simulation, Liverpool, L8 1XE UK

**Keywords:** Electrical and electronic engineering, Computational science

## Abstract

The wireless network data are a feasible way to understand the user behavior in a given environment and may be utilized for analysis, prediction and optimization. On the other hand, datasets from wireless service providers are not publicly available, and obtaining a dataset in real time is challenging. In this work, we present a 5G dense deployment dataset obtained from the Liverpool City Region High Density Demand (LCR HDD) project. The project involves network deployment and assessment at Salt & Tar and the ACC Arena event venues located in the city of Liverpool. Digital twin technology is considered to generate the dataset, which is inputted to a system level simulator for data modeling and analysis. The data set consists of 3, 000 users in the Salt & Tar venue and 12, 000 users in the ACC Arena venue with features including users’ position, traffic type, Radio Unit (RU) association, Signal to Interference and Noise Ratio (SINR), Physical Resource Blocks (PRB), throughput, Block Error Rate (BLER), and a total length of 10, 000 samples. The dataset is validated through experimental measurements and is released in a simple format for easy access.

## Background & Summary

The 5th Generation (5G) subscriptions are increasing exponentially, and it is expected that by the end of 2027, 5G will be the dominant mobile technology and will overtake 4th Generation (4G) subscriptions. Specifically, by 2030, global 5G subscriptions are forecast to reach 6.3 billion, which includes 3.6 billion 5G Standalone (SA) subscriptions worldwide^1^. This increasing demand requires dense network deployment, real-time monitoring, and automated/dynamic optimization. To help facilitate this, the devices connected to the network also generate a large amount of data and may be utilized for network optimization and data-driven control and management^2^. In this context, Artificial Intelligence (AI) and Machine Learning (ML) techniques are creating advancements in many industrial applications, including wireless communications, and require datasets for generalization, training, and implementation. On the other hand, collecting a dataset in real time from physical measurements to upper-layer network analysis is expensive, and the datasets from cellular operators are not publicly available due to privacy concerns and legal issues^3^.

Several researchers collected wireless datasets that are already available publicly^4–12^. Liatifis *et al*.^4^ presented a 5G New Radio (NR) coverage expansion dataset in the context of the NANCY project. The dataset consists of the following two experiments: (a) User Equipment (UE) is directly connected to a Base Station (BS); and (b) an intermediate node is employed between the BS and UE, and acts as a relay. The experiment considered two 5G BSs and two 5G UE, and the dataset consists of iPerf3 (both TCP and UDP) and video streaming data. Zhnag J.^5^, presented a 6th Generation (6G) and beyond network deployment dataset. The author introduced a novel Deep Reinforcement Learning (DRL) approach for integrated access and backhaul network planning while considering urban dynamics and constraints. Authors in^6^ published a time series of a received signal power dataset. The measurements were carried out in an empty hall with dimension 7.5 m  × 4.5 m  × 3 m, simultaneously capturing blockage, micro-mobility, and beam tracing. City-wide measurements of 4G and 5G cellular networks in low and mid bands were performed by Rochman *et al*.^7^. The measurements were carried out in Chicago and Minneapolis to analyze the throughput performance improvement and different aspects of 5G advancements. Choi *et al*.^3,8^, published a Machine Learning (ML) based 5G traffic generation using open datasets. The dataset was generated using two types of ML models and a wide range of applications, from video to the metaverse. The dataset is publicly available at IEEE DataPort^8^. Liu *et al*.^9^, presented an in-depth ten-month measurement study of 5G considering three US top operators (AT&T, Verizon, and T-Mobile). The authors quantified the 5G availability, coverage, performance issues, and their root causes. The authors developed a patch solution, 5GBoost to fix the identified legacy 5G operation issues. A 5G trace dataset was collected from an Irish mobile operator by Raca *et al*.^10^. The dataset was collected with two mobility patterns, static and moving in a car, considering video streaming and file download applications. The dataset consists of channel, context, cell-related matrices, and throughput information. The radio, positioning, throughput, and beam switch log data set was collected in Albarog University Smart Production Lab^11^. Shah *et al*.^12^ collected the wireless channel measurement between two simulated devices using the Vienna 5G Link Level Simulator^13^ in four different wireless environments. The data consist of channel state information (CSI), signal magnitude, phase variations, enabling the evaluation of symbol disagreement rate (SDR), bit disagreement rate (BDR), and key error rate (KER), and may be utilized for wireless security. A summary of the major datasets considered from the state of the art is presented in Table 1, which includes the considered mobile network technology, the nature of the data, and a summary of the work for each referenced paper.

**Table 1:** Overview of selected related datasets.

Ref.	Technology in Focus	Data	Work Summary
Vasilakos’ 20^2^	4G/5G	• Radio Access Network (RAN) monitoring data from Medium Access Control (MAC), Radio Link Control (RLC) and Packet Data Convergence Protocol (PDCP) alyers	• ElasticSDK, a software development kit for monitoring, control, and management in Software-Defined Networking (SDN) enabled 5G network• One user’s data with more than 100 measurements in a JSON format
Choi’ 23^3,8^	5G	• Streaming, video conferencing, metaverse and online gaming dataset	• Collected 328 hours long dataset• Presented two ML models for 5G traffic generation• The model generated 75 minutes traffic in less than 1 second
Liatifis’ 23^4^	5G	• Network resource utilization dataset	• 5G New Radio (NR) coverage expansion and network’s stability• Two 5G base stations and two 5G users are considered to generate resource utilization through data-intensive streaming
Ershova’ 24^6^	6G	• Received Signal Power (RSP) dataset	• Time series of RSP, considering blockage and micro mobility• Traces can be utilized for designing beam tracking algorithms and statistical analysis
Rochman’ 24^7^	5G and next generation networks	• Dataset consists of timestamp, position, operator, Synchronization Signal Block (SSB), Reference Signal Received Power (RSRP) and other features	• 4G/5G cellular network performance measurement in 1 ~ 6 GHz band• Next generation cellular networks design considers wider channel, dense network deployment, better signal strength, and no more than 4 MIMO layers
Liu’ 23^9^	4G and 5G	• 5G availability, coverage and Reference Signal Received Power/Reference Signal Received Quality dataset	• Data collected with AT&T, Verizon and T-Mobile network operators• Designed a patch (5GBoost) to enhance the radio resource control
Raca’ 20^10^	5G	• Throughput, channel and context information	• 5G dataset collected from a major Irish mobile operator• Dataset collected from static and car mobility patterns, considering video streaming and download applications
Tarrías’ 24^11^	5G	• Radio, positioning, throughput and beam switching log	• Dataset collected using an automated guided vehicle in Aalborg University’s smart production lab• Beam switching based on RSRP and position information
Shah’ 25^12^	5G	• Channel state information (CSI), signal magnitude and phase variations	• Dataset collected considering two device using Vienna 5G simulator• May be utilized for physical-layer security and cryptographic applications evaluation
This work	5G and 6G	• Latitude, longitude, traffic type, Signal to Interference and Noise Ratio (SINR) and throughput information	•Dataset for 5G and 6G network dense deployment•Digital twin and 5G simulator based dataset•Dataset for model creation, machine learning, quality prediction

However, the datasets collected^4–12^ may not be utilized for high-density network deployment and optimization, as they do not consider 5G dense deployments i.e. areas with a large number of users and devices. This work goes beyond the state of the art by presenting a dataset obtained from the LCR HDD project, which sought to demonstrate the benefits of Open Radio Access Network (O-RAN) technology in optimizing real-world performance in high-connectivity environments located in the city of Liverpool^14^. Specifically, the project aimed to deploy and assess 5G networks supported by O-RAN in two high-density venues, Salt & Tar and ACC Arena, located in Liverpool. For network modeling, a patterns of life digital twin model was created for both scenarios to analyze the users’ mobility and traffic type. The generated user position and traffic patterns were then inputted to an O-RAN-based 5G system-level simulator implemented in the context of the LCR HDD project to optimize the network performance. In summary, the generated dataset includes user mobility, traffic type, Radio Unit (RU) association, throughput, Signal to Interference and Noise Ratio (SINR), Physical Resource Blocks (PRB), and Block Error Rate (BLER), which are made publicly available. The generated dataset may be utilized for prototyping, predictive analysis, real network deployment and evaluating the performance of networks in high-density deployment.

## Methods

The LCR HDD project provides a private O-RAN-based deployment to support 5G networks in real-world high-density environments, and this was first trialed at Salt & Tar’s music festival, a four-day festival held in August 2024 that attracted 12, 000 fans to Bootle’s outdoor venue^15^. The main project’s objectives revolve around the ideas of optimizing the network performance through the use of O-RAN, while guaranteeing that the user experience is maintained as far as possible. To this end, we designed and developed the O-RAN based Liverpool 5G (L5G) simulator to evaluate the optimizations proposed in the project within a simulated environment first, replicating the venues prior to real-world implementation. Currently, the L5G simulator serves as a powerful tool that extends beyond the scope of the LCR HDD project, enabling the development and testing of 5G and Beyond 5G (B5G) networks based on the O-RAN architecture in a controlled simulation environment.

**Fig. 1:**
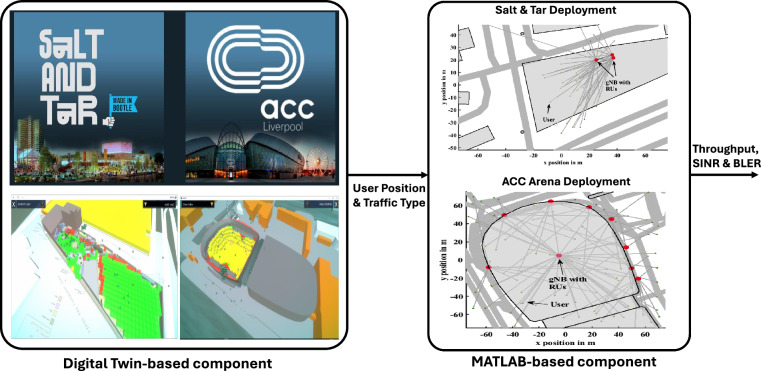
L5G Simulator: The simulator consists of two main components, a digital twin-based model of the venues and a Matlab-based simulator of the deployment.

The L5G simulator consists of two main components illustrated in Fig. 1: a digital twin-based model of the venues and a Matlab-based simulator of the deployments. The digital twin component is developed by CGA simulation^16^, a virtual simulation capable of solving real-world problems based on realistic user behaviour in terms of mobility and data traffic, achieved from real events. The digital twin provides a virtual representation of venues, where various scenarios can be tested, including network congestion, coverage gaps, and optimal cell positioning. This model allows for (i) Real-time visualisation of network performance; (ii) Automated testing of xApps under different venue conditions; and (iii) Generation of synthetic data for AI-driven analytics and network optimisation. The simulation adopts a dual-coordinate reference frame in which all agent motion and radio-access calculations are performed in Unity’s Cartesian space, while geographic reporting is expressed in World Geodetic System 1984 (WGS-84) lat/long/altitude. A single geodetic origin—captured at start-up from the Mapity singleton—anchors the two frames. All subsequent conversions apply a fixed linear offset to the vector position. It is the positional offset of the two worlds without accounting for earth curvature or higher-order ellipsoidal terms; thus, positional error grows with distance from the origin but is deemed negligible for the intended local-area (city-scale) studies. Terrain elevation is held constantly, so vertical variation is ignored except where an external system injects altitude into the conversion utility. User Equipment (UE) behaviour is modelled with a Poisson process. This is achieved by abstracting as a continuous-time, finite-state Markov process over enumerated Mobile usage classes (off, idle, constant rate, video, HTTP and Gaming). The wait time in each usage state is sampled from an exponential distribution with a mean specified according to the current activity (e.g. probability of each possible mobile phone use is different when in a queue to the toilet/food stand than when dancing); this embeds the assumption of memoryless dwell times and independent device trajectories. The transition probabilities are conditioned on the agent’s current activity within the simulation environment, as defined by a corresponding look-up table associated with that specific activity. In the absence of an activity component, a global default table is utilized. These probabilities have been derived from general observational data collected across a range of events with a random seed, which means that results are non-deterministic across simulation runs. Since radio-access infrastructure is topologically static, the spatial coverage is discretised into an orthogonal lattice of event space. During each frame, an agent is associated with the single nearest Mast (cell) that also owns the coverage box currently containing the agent’s position; consequently, connection choice is governed solely by Euclidean distance and Boolean range checks. Mast range can be modified at runtime, but the user (or an external optimisation routine) must explicitly call the recalculate coverage function; otherwise, the change has no effect, placing a procedural constraint on dynamic experiments.

The Matlab-based component is developed from the Vienna 5G System Level (SL) Simulator^13^, which has the capability to mimic buildings, streets, radio environment conditions, such as path loss and shadowing, developed according to real-world scenarios based on data available in the OpenStreetMap (OSM) database. The L5G Simulator extends and enhances this by implementing: 1) the deployments identified for the LCR HDD project and the 5G RUs based on the project specifications; 2) a new function, which calculates the throughput based on the 5G Orthogonal Frequency Division Multiplexing (OFDM) and 5G specifications^17^; and 3) a module that simulates O-RAN-based components^18^. Specifically, we designed and developed a module that implements a Near-Real Time RAN Intelligence Controller (Near-RT RIC) and the interface that allows the communication between the RIC and the RUs to gather information from the radio environment. We considered nine RUs placed according to our physical deployment in Liverpool’s Salt & Tar area outdoor event venue, and thirty three RUs placed according to the deployment in Liverpool’s ACC Arena indoor event venue, i.e., twenty four RUs located around the main scenario, and nine RUs in the roof of the main scenario as illustrated in Fig. 1. Both deployments consider 3.8 GHz operating frequency, 100 MHz bandwidth, 4 × 4 MIMO, 49 dBm Equivalent Isotropic Radiated Power (EIRP), 12 dBi antenna directivity, and 256 QAM MCS-Table. The subcarrier spacing is 30 KHz, 12 subcarriers in the resource grid with 14 symbols are considered, 2 symbols are reserved for Control Resource Set (CORESET), Channel State Information Reference Signal (CSI-RS) is transmitted in different slots of each RU, the Cyclic Prefix (CP) duration is 4.69*μ*s, and the CP ratio is 10.12%. The parameters used during simulation are given in Table 2. During the experiments, we considered 3, 000 users in the Salt & Tar event venue and 12, 000 users in ACC Arena event venue. The users are both mobile and stationary, running realistic applications such as video streaming, web browsing, gaming, and Voice over IP (VoIP). The digital twin-based component generates the user’s position and application data for 3, 000 users in Salt & Tar and 12, 000 users in ACC Arena for 10, 000 timestamps generated every 1 second in Salt & Tar and every 3 seconds in ACC Arena. These data can be input into the MATLAB-based component, which associates each user at each timestamp to the RU providing the highest Received Signal Strength (RSS). Moreover, after the users connect to the RU, the Near-RT RIC implemented in the MATLAB-based component can gather information from the radio environments and compute key performance parameters based on the input generated by the digital twin-based component, including downlink (DL) and uplink (UL) SINR (dB), user throughput (Mbps), PRB utilization, and BLER at every timestamp for each user, considering their current position and application. On the other hand, due to the complexity of the scenario and to the large file size, the data is divided into multiple files, with each file containing 500 users’ data. Further details on the datasets will be provided in the next section.

**Table 2:** Simulation Parameters.

Parameter	Values
Operating Frequency	3.8 GHz
Bandwidth	100 MHz
MIMO	4 × 4
EIRP	49 dBm
Antenna Gain	12 dBi
MCS Table	256 QAM
Subcarrier Spacing	30 KHz
CP duration	4.69 *μ*s

## Data Records

**Fig. 2:**
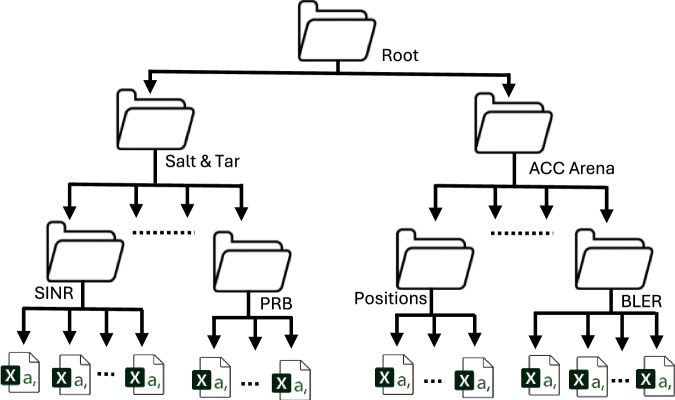
The dataset is organized into compressed folders for Salt & Tar and ACC Arena event venues. The dataset includes users’ positions information, application running, downlink (DL) and uplink (UL) SINR (dB), user throughput (Mbps), PRB utilization, and BLER at every timestamp for each user.

We provide the described dataset via the LJMU data repository (https://opendata.ljmu.ac.uk/id/eprint/236/)^19^. The dataset is organized into compressed folders containing comma-separated values (CSV) files for each of the venues. The overview of the folder hierarchy is shown in Fig. 2, and the details of the files and features are as follows:

### Salt & Tar dataset

The folder Salt & Tar dataset consists of 3, 000 users data with 10, 000 samples. The folder is further divided into sub-folders, and the features of the datasets are the user’s position, traffic type, generated by the digital twin-based component, and RU association, throughput, SINR UL, SINR DL, PRB, and BLER generated by the MATLAB-based component. The description of the fields in the file is:

Sample User Positions Data (CSV): The file contains the time, positions, and traffic type information for each user and time stamp. Due to the large file size, the data is divided into six files, with each file containing 500 users’ data. There is a total of 25, 010, 000 data values in each file, and the summary of data in all the files is given in Table 3. The columns in the files include the following information:

- Time stamp: The time stamp information is represented in seconds.
- Entity stats id: The id of the user. The id 0 corresponds to user 1, id 499 corresponds to user 500, and id 2999 corresponds to user 3000.
- Latitude: The user position information in terms of Latitude.
- Longitude: The user position information in terms of Longitude.
- Altitude: The user position information in terms of Altitude.
- Mobile state: The information about user traffic. Specifically, this column contains values between 0 ~ 5. The values {0, 1, 2, 3, 4, 5} correspond to {off, idle, constant rate, video, gaming, http}, respectively.

**Table 3:** Users’ latitude, longitude, altitude, and traffic type information for each user and time stamp in Salt & Tar event venue.

Time stamp (s)	Entity stats id	Latitude	Longitude	Altitude	Mobile state	Entity stats id	...	Entity stats id	...	Mobile state
**File 1 (Positions_Salt_Tar_UE_Id_0_499)**										
403	0	53.45038973	−2.991965675	0.00634267	5	1	...	499	...	1
404	0	53.45038973	−2.991965675	0.00634267	5	1	...	499	...	1
...	...	...	...	...	...	...	...	...	...	...
...	...	...	...	...	...	...	...	...	...	...
10415	0	53.45038973	−2.991965675	0.00634267	5	1	...	499	...	1
**File 2 (Positions_Salt_Tar_UE_Id_500_999)**										
403	500	53.45041628	−2.991903802	0.00635264	2	501	...	999	...	1
404	500	53.45041628	−2.991903802	0.00635264	2	501	...	999	...	1
...	...	...	...	...	...	...	...	...	...	...
...	...	...	...	...	...	...	...	...	...	...
10415	500	53.45041628	−2.991903802	0.00635264	2	501	...	999	...	1
**:**										
**:**										
**File 6 (Positions_Salt_Tar_UE_Id_2500_2999)**										
403	2500	53.4504219154321	−2.99207134	0.006306032	1	2501	...	2999	...	1
404	2500	53.4504219154321	−2.99207134	0.006306032	1	2501	...	2999	...	1
...	...	...	...	...	...	...	...	...	...	...
...	...	...	...	...	...	...	...	...	...	...
10415	2500	53.450312988259	−2.992633585	0.006339582	4	2501	...	2999	...	1

The same values are repeated across 500 users in each of the six files.

RU Association Data (CSV): There are nine RUs deployed in the Salt & Tar deployment. The file contains the user RU association information corresponding to each position. The summary of data in this file is given in Table 4. The columns in the files include the following information:

- Time stamp: The time stamp information is represented in seconds.
- Entity stats id 0: RU association of the first user.
- Entity stats id 1: RU association of the second user.
- … … …
- … … …
- Entity stats id 499: RU association for user 500.

**Table 4:** Users’ RU Association for each time stamp in Salt & Tar.

File 1 (RU_UE_Id_0_499)					
Time stamp (s)	Entity stats id 0	Entity stats id 1	Entity stats id 2	...	Entity stats id 499
403	5	2	2	...	2
404	5	2	2	...	2
...	...	...	...	...	...
...	...	...	...	...	...
10415	5	2	2	...	2
**:**					

File 6 (RU_UE_Id_2500_2999)					
Time stamp (s)	Entity stats id 2500	Entity stats id 2501	Entity stats id 2502	...	Entity stats id 2599
403	8	2	9	...	3
404	2	2	9	...	9
...	...	...	...	...	...
...	...	...	...	...	...
10415	6	2	3	...	3

SINR DL Data (CSV) and SINR UL Data (CSV): The files contain the user downlink and uplink SINR corresponding to each position. The summary of data in this file is given in Tables 5 and 6. The columns in the files include the following information:

- Time stamp: The time stamp information is represented in seconds.
- Entity stats id 0: SINR for first user.
- Entity stats id 1: SINR for second user.
- … … …
- … … …
- Entity stats id 499: SINR for user 500.

**Table 5:** SINR DL(dB) for each time stamp in Salt & Tar.

Time stamp (s)	Entity stats id 0	Entity stats id 1	Entity stats id 2	...	Entity stats id 499
403	−5.726239239	−12.18296599	−7.954882305	...	−8.214111167
404	−5.359294685	−10.51245506	−17.01227468	...	−4.941011248
...	...	...	...	...	...
...	...	...	...	...	...
10415	−9.15904679	−3.670438516	−7.33994333	...	−3.485268616

**Table 6:** SINR UL(dB) for each time stamp in Salt & Tar.

Time stamp (s)	Entity stats id 0	Entity stats id 1	Entity stats id 2	...	Entity stats id 499
403	−4.489734743	−11.30203544	−7.018842523	...	−17.52410027
404	−5.027732685	−8.226217875	−18.93171384	...	−9.204608332
...	...	...	...	...	...
...	...	...	...	...	...
10415	−8.851117553	−3.602085854	−5.998839989	...	−2.827736239

Throughput Data (CSV): The file contains the user throughput corresponding to each position. The summary of data in this file is given in Table 7. The columns in the files include the following information:

- Timestamp: The time stamp information is represented in seconds.
- Entity stats id 0: Throughput for first user.
- Entity stats id 1: Throughput for second user.
- … … …
- … … …
- Entity stats id 499: Throughput for user 500.

**Table 7:** Throughput (Mbps) for each time stamp in Salt & Tar.

Time stamp (s)	Entity stats id 0	Entity stats id 1	Entity stats id 2	...	Entity stats id 499
404	0	0.296	0	...	0
...	...	...	...	...	...
5648	0	0.776	2.288	...	0.736
...	...	...	...	...	...
10415	0	0.296	0	...	0

Physical Resource Block (PRB) and Block Error Rate (BLER) (CSV): These files contain the PRB and BLER data. The file columns are structured similarly to the SINR and throughput data files. The summary of the PRB and BLER data files is given in Tables 8 and 9, respectively. The data value Zero in the dataset means the user is not active, or it is not scheduled for transmission or reception at that time.

**Table 8:** Physical Resource Block (PRB) for each time stamp in Salt & Tar.

Time stamp (s)	Entity stats id 0	Entity stats id 1	Entity stats id 2	...	Entity stats id 499
404	0	16	0	...	0
...	...	...	...	...	...
5648	0	16	20	...	38
...	...	...	...	...	...
10415	0	16	0	...	0

**Table 9:** Block Error Rate (BLER) for each time stamp in Salt & Tar.

Time stamp (s)	Entity stats id 0	Entity stats id 1	Entity stats id 2	...	Entity stats id 499
403	0	0	9.41e-07	...	0
...	...	...	...	...	...
5648	0	0	9.41e-07	...	0.1966755
...	...	...	...	...	...
10415	0	0	0	...	0

#### ACC Arena dataset

This folder contains the users’ data obtained at the ACC Arena venue. The folder is further divided into sub-folders, and the features of the datasets are the user’s position, traffic type, generated by the digital twin-based component, and RU association, throughput, SINR UL, SINR DL, PRB, and BLER generated by the MATLAB-based component. The overview of the folder hierarchy is shown in Fig. 2, and the arrangement of the files is similar to Salt & Tar Venue data files. The dataset consists of 12, 000 users’ data with 10, 000 samples. Due to the large file size, the data is divided into twenty-four files, each file containing 500 users’ data. The file Sample User Positions Data (CSV) contains features (time, positions, and traffic type) similar to Salt & Tar Venue, and the summary of position data is given in Table 10. Moreover, the RU association, SINR DL, SINR UL, Throughput, PRB, and BLER (CSV) files are arranged in a format similar to Tables 4, 5, 6, 7, 8, and 9, respectively, and contain information similar to Salt & Tar Venue data files.

**Table 10:** Users’ latitude, longitude, altitude, and traffic type information for each user and time stamp in ACC Arena event venue.

Time stamp (s)	Entity stats id	Latitude	Longitude	Altitude	Mobile state	Entity stats id	...	Entity stats id	...	Mobile state
**File 1 (Positions_Acc_Arena_UE_Id_0_499)**										
1212	0	53.395083	− 2.992707132	3.581008991	2	1	...	499	...	1
1215	0	53.395083	− 2.992707132	3.581008991	2	1	...	499	...	1
...	...	...	...	...	...	...	...	...	...	...
...	...	...	...	...	...	...	...	...	...	...
33968	0	53.395083	− 2.992707132	3.581008991	2	1	...	499	...	1
**File 2 (Positions_Acc_Arena_UE_Id_500_999)**										
1212	500	53.3978678	− 2.992795298	3.661016858	2	501	...	999	...	2
1215	500	53.3978678	− 2.992795298	3.661016858	2	501	...	999	...	2
...	...	...	...	...	...	...	...	...	...	...
...	...	...	...	...	...	...	...	...	...	...
33968	500	53.3978678	− 2.992795298	3.661016858	2	501	...	999	...	2
**:**										
**:**										
**File 24 (Positions_Acc_Arena_UE_Id_11500_11999)**										
1212	11500	53.40157349	− 2.995471567	0.025163376	1	11501	...	11999	...	1
1215	11500	53.40157349	− 2.995471567	0.025163376	1	11501	...	11999	...	1
...	...	...	...	...	...	...	...	...	...	...
...	...	...	...	...	...	...	...	...	...	...
10415	11500	53.40157349	− 2.995471567	0.025163376	1	11501	...	11999	...	5

## Technical Validation

In order to validate the dataset, we performed several real-world tests in the LCR HDD project venues. In this paper, we illustrate one test for each venue. The first test is performed in the Salt & Tar venue to validate the throughput. The test involved four UEs using full-buffer with iPerf or Spirent TestCenter Applications for 10 minutes, located at approximately 25 meters from one of the RUs located in Salt & Tar. The same test is repeated in the simulator by inputting the user’s real-time location (longitude and latitude) and application type obtained from the experiment. The average throughput achieved during the experiment is 235.7 Mbps, and the throughput obtained by the simulator is 244.36 Mbps. The difference between experiment and simulation is 3.5%, and it is less than 10%, which was expected due to manufacturing tolerance^20^.

The second test was performed by LJMU volunteers at the ACC Arena using the RU that serves the main scenario. Fifty users browsed different applications, such as YouTube and TikTok, while moving throughout the main scenario, and then they sat in the Arena around the scenario for approximately one hour. The average PRB obtained through real-time experiment and simulation are 126 and 130, respectively. Therefore, the test validates the dataset, which is publicly available and can be used for network analysis, optimization, and prediction.

## Usage Notes

The generated dataset may be utilized in real network deployment and industries such as machine learning, research, and development as follows:

- Machine Learning based modeling: The dataset may be utilized for network performance predictive analysis and to train the generative model to produce new data.
- Research and Development: Measurement may be used for another deployment environment analysis, experimentation, and results validation. Moreover, the dataset may be utilized to depict the custom behavior of new software in a simulation.
- Prototyping and training: Prototyping the performance in 5G network topologies and educating on the performance of 5G networks in high-density deployments. The data set collected in Salt & Tar and the ACC Arena may be utilized for generalization of outdoor event venues and indoor event venues, respectively.

In addition, the proposed folder-based structure and dataset in a single format (.CSV files) make it easier for the community to use it and extend this dataset by considering more scenarios and different configurations. We do not provide code to extract the data; however, that can be implemented using standard tools such as MATLAB/Pandas.

## Data Availability

The complete dataset is available as a zip file at the LJMU repository (https://opendata.ljmu.ac.uk/id/eprint/236/)^19^. We also created a mirror of the dataset, and it is available online at the Zenodo repository (https://zenodo.org/records/16565029).

## Code availability

The dataset is generated through the Liverpool 5G (L5G) simulator, and the work is part of the Liverpool City Region High-Density Deployment Project. The L5G simulator consists of two main components: (i) a digital twin-based model of the venues and (ii) a Matlab-based simulator of the deployments. The digital twin component is developed by CGA simulation^16^ and is available online at (https://opendata.ljmu.ac.uk/id/eprint/251/)^21^. The MATLAB-based deployment simulator is built upon the Vienna 5G System Level (SL) Simulator and is proprietary to Liverpool John Moores University. Moreover, the original Vienna 5G SL Simulator^13^ is available for academic use after getting a free academic license from its authors. One may follow the steps given in the guidelines available online at (https://opendata.ljmu.ac.uk/id/eprint/251/)^21^ to configure the Vienna 5G SL simulator and to regenerate the result.
